# Insufficiencies in sensory systems reweighting is associated with walking impairment severity in chronic stroke: an observational cohort study

**DOI:** 10.3389/fneur.2023.1244657

**Published:** 2023-11-03

**Authors:** Oluwole O. Awosika, Amanda Garver, Colin Drury, Heidi J. Sucharew, Pierce Boyne, Sarah M. Schwab, Emily Wasik, Melinda Earnest, Kari Dunning, Amit Bhattacharya, Pooja Khatri, Brett M. Kissela

**Affiliations:** ^1^Department of Neurology and Rehabilitation Medicine, College of Medicine, University of Cincinnati, Cincinnati, OH, United States; ^2^Department of Emergency Medicine, University of Cincinnati, Cincinnati, OH, United States; ^3^Department of Rehabilitation, Exercise and Nutrition Sciences, College of Allied Health Sciences, University of Cincinnati, Cincinnati, OH, United States; ^4^EDDI Lab—Early Detection of Degenerative Disorders and Innovative Solutions, Department of Environmental Health, University of Cincinnati, Cincinnati, OH, United States

**Keywords:** walking, stroke, balance, sensorineural integration, sensory reweighting, gait, outcome measures, stroke recovery

## Abstract

**Background:**

Walking and balance impairment are common sequelae of stroke and significantly impact functional independence, morbidity, and mortality. Adequate postural stability is needed for walking, which requires sufficient integration of sensory information between the visual, somatosensory, and vestibular centers. “Sensory reweighting” describes the normal physiologic response needed to maintain postural stability in the absence of sufficient visual or somatosensory information and is believed to play a critical role in preserving postural stability after stroke. However, the extent to which sensory reweighting successfully maintains postural stability in the chronic stages of stroke and its potential impact on walking function remains understudied.

**Methods:**

In this cross-sectional study, fifty-eight community-dwelling ambulatory chronic stroke survivors underwent baseline postural stability testing during quiet stance using the modified Clinical test of Sensory Interaction in Balance (mCTSIB) and assessment of spatiotemporal gait parameters.

**Results:**

Seventy-six percent (45/58) of participants showed sufficient sensory reweighting with visual and somatosensory deprivation for maintaining postural stability, albeit with greater postural sway velocity indices than normative data. In contrast, survivors with insufficient reweighting demonstrated markedly slower overground walking speeds, greater spatiotemporal asymmetry, and limited acceleration potential.

**Conclusion:**

Adequate sensory system reweighting is essential for chronic stroke survivors’ postural stability and walking independence. Greater emphasis should be placed on rehabilitation strategies incorporating multisensory system integration testing and strengthening as part of walking rehabilitation protocols. Given its potential impact on outcomes, walking rehabilitation trials may benefit from incorporating formal postural stability testing in design and group stratification.

## Introduction

Post-stroke walking impairment significantly reduces functional ambulatory independence and is a significant cause of worldwide morbidity and mortality (1, 2). Commonly referred to as the sixth vital sign (3), walking speed is a crucial measure of post-stroke walking impairment, a determinant of functional independence levels, and a predictor of life expectancy (4). In the same vein, stroke survivors often experience balance impairment as a functional sequela and are at an increased risk of falls throughout the continuum of recovery during standing and walking (5, 6). Consequently, stroke survivors with balance impairment often experience low confidence in walking—leading to the loss of life roles, social isolation, dependency, a sedentary lifestyle, and an increased risk of falls, fractures, and secondary medical complications (7). While walking and standing balance have inherent functional and biomechanical differences, one of many key similarities is their dependence on postural stability (8, 9).

Postural stability is the ability to maintain the center of gravity inside one’s base of support through discrete synergies of trunk and leg muscles and is activated both in quiet stance and during motion (10–12). Evidence suggests that the visual, somatosensory (proprioception, joint, cutaneous), and vestibular systems centrally mediate sensorineural integration and processing, and that this sensory integration is critical for maintaining static and dynamic postural stability (8). While the preferred sensory system for maintaining postural stability in non-disabled adults is somatosensory input; the visual and vestibular centers are believed to play complementary roles in resolving conflicting sensory input (13). The subconscious ability to enhance the influence of one type of sensory input to compensate for a decrease or absence of information from another sensory center is termed “sensory reweighting (14, 15).” This phenomenon enables neurologically intact persons to maintain postural stability during visual or somatosensory deprivation. However, after neurologic injury, sensory reweighting is often impaired in the acute stages of stroke and is believed to improve over time—thereby improving motor control and postural stability (16–18). Therefore, it is possible that insufficiencies in sensory reweighting could lead to sustained postural instability, increase the risk of falls, and impact walking performance.

While progress has been made over the past three decades to characterize the inner workings of sensory system adaptations post-stroke (19–23), critical knowledge gaps remain. For example, though stroke survivors are believed to undergo reweighting (14, 24), the extent to which this process sufficiently facilitates postural stability is unknown. In addition, the downstream impact of postural stability on walking capacity measures has been under-reported. Furthermore, the most essential, if any, of the three sensory centers remains unsettled. For example, past work by Bonan and colleagues examined postural stability during quiet stance in 40 chronic stroke survivors and found that stroke survivors relied excessively on visual input to maintain posture (25, 26). In contrast, using a similar study posturography paradigm, Oliveira et al. reported that somatosensation was more critical for postural stability in their cohort (*N* = 21) (14). Yet, others have reported that visual input and somatosensation play equal roles (5, 27–29). The variations in viewpoints may, in part, result from the vast heterogenicity in baseline comorbidities, stroke lesion size, location, chronicity, the trajectory of recovery, variations in rehabilitation interventions, and social determinants such as access to rehabilitation and community involvement. Nonetheless, further investigations into reweighting and sensorineural integration and its impact on walking function in the chronic stages of stroke recovery are essential to advance the collective understanding of factors influencing balance and walking outcomes.

Hence, the objectives of this study were: (1) to characterize sensory reweighting in cohort of chronic stroke survivors (≥6 months post-stroke) and (2) to determine the relationship between postural stability and walking speed. We hypothesized that chronic stroke survivors would have greater postural instability across all four conditions of the modified Clinical test of Sensory Interaction in Balance (mCTSIB) compared to normative data. Second, participants with the most significant postural instability or the overt loss of balance (non*-completion*) would demonstrate greater walking impairment (decreased walking speed and acceleration potentials and increased spatiotemporal asymmetry).

## Methods

### Participants

Fifty-eight community-dwelling, ambulatory chronic stroke survivors (mean age: 56.9 ± 8.79, Female = 29) who had undergone baseline testing in two walking rehabilitation trials (NCT04553198 and NCT04721860) at the University of Cincinnati Neurorecovery Lab from August 2020 to February 2023 were included in this study. According to the Declaration of Helsinki recommendations, all participants provided written informed consent prior to enrollment. Inclusion criteria were: 18–80 years of age, residual walking impairment secondary to ischemic/hemorrhagic stroke(s) and ambulating at least 10 meters without a walker. All participants were able to walk independently without assistance of another person (with or without orthosis) or with an assistive device (cane, quad cane). In addition, all participants were expected to have abstained from formal physiotherapy and botulinum toxin injections at least 2 weeks before the screening visit (30). Exclusion criteria were unstable cardiovascular status precluding participation in a moderate-high intensity exercise, an adverse health condition that might affect walking capacity (other than stroke), severe lower extremity spasticity (modified Ashworth > 2/4), or significant language barrier which may interfere with the ability to follow instructions during testing. The baseline demographics of study participants are highlighted in Table 1. For the purposes of this study, it is worth noting that the current recovery and rehabilitation guidelines identify chronic stroke as individuals who are ≥6 months (without an upper limit), with the idea that most of the recovery will occur between the first 6 months, and to a lesser extent thereafter (31).

**Table 1:** Baseline demographics and characteristics of study participants.

Study ID	Age (years)	Sex (M/F)	assistive device	AFO (Y/N)	Stroke type (I/H)	Lateralization (L/R/BL)	Hemispheric/brain stem-cerebellum	Time since stroke (months)	Mini mental status exam
1	58	M	None	N	I	L	Hemispheric	31	28
2	60	M	None	N	I	L	Brainstem	12	30
3	52	F	None	Y	H	R	Hemispheric	69	30
4	57	F	Cane	N	I	R	Hemispheric	42	30
5	58	M	None	N	I	L	Hemispheric	23	28
6	49	F	None	N	I	R	Hemispheric	10	30
7	56	F	None	N	I	R	Hemispheric	11	30
8	65	F	None	N	I	BL	Hemispheric	192	24
9	54	F	None	N	H	L	Brainstem	15	30
10	53	F	None	N	I	L	Hemispheric	38	28
11	59	M	None	Y	I	L	Hemispheric	91	30
12	66	M	None	N	I	L	Hemispheric	260	25
13	60	M	None	N	I	R	Hemispheric	55	30
14	67	M	None	N	I	R	Hemispheric	18	30
15	48	F	None	Y	I	R	Hemispheric	44	30
16	59	M	None	Y	I	R	Hemispheric	49	30
17	41	F	None	N	I	BL	Mixed	99	28
18	58	F	None	N	I	L	Hemispheric	12	30
19	63	F	Cane	N	I	L	Hemispheric	25	30
20	57	M	None	N	I	L	Brainstem	7	30
21	59	F	None	N	I	L	Hemispheric	6	30
22	41	F	None	N	I	L	Hemispheric	38	29
23	57	M	None	N	I	L	Hemispheric	85	29
24	63	M	Cane	N	I	L	Mixed	7	23
25	67	M	None	N	I	L	Hemispheric	180	23
26	71	M	None	N	I	R	Hemispheric	50	30
27	50	M	None	Y	I	L	Hemispheric	11	30
28	67	M	None	Y	I	L	Hemispheric	63	30
29	55	F	None	Y	I	R	Hemispheric	68	30
30	55	M	Cane	N	I	R	Hemispheric	9	29
31	42	F	None	N	I	R	Hemispheric	27	30
32	73	M	Quad Cane	Y	I	R	Hemispheric	10	30
33	68	F	None	N	I	R	Mixed	10	30
34	51	M	Cane	N	I	R	Hemispheric	6	30
35	54	F	Cane	Y	I	L	Hemispheric	34	24
36	53	F	Quad Cane	N	I	R	Hemispheric	47	24
37	42	F	Cane	N	H	L	Hemispheric	30	24
38	43	F	None	N	H	R	Hemispheric	21	29
39	52	F	None	N	H	L	Hemispheric	15	30
40	44	F	None	N	I	R	Hemispheric	11	30
41	42	M	Cane	N	H	L	Hemispheric	45	30
42	65	M	Quad Cane	N	H	R	Hemispheric	95	30
43	53	M	Quad Cane	N	H	BL	Brainstem	45	30
44	67	M	Quad Cane	N	H	L	Brainstem	38	29
45	64	M	Cane	N	I	R	Hemispheric	300	30
46	55	M	Cane	Y	H	R	Hemispheric	59	30
47	57	F	None	N	I	L	Brainstem	19	30
48	71	M	Cane	Y	I	R	Hemispheric	47	29
49	68	F	Cane	Y	I	R	Hemispheric	42	30
50	58	M	Quad Cane	Y	I	R	Hemispheric	6	29
51	48	F	Quad Cane	N	H	L	Hemispheric	14	24
52	50	M	Quad Cane	Y	H	L	Hemispheric	44	23
53	64	M	Quad Cane	Y	H	L	Hemispheric	7	28
54	56	M	Quad Cane	N	I	R	Hemispheric	24	29
55	61	F	Quad Cane	N	I	BL	Hemispheric	24	30
56	79	F	Quad cane	N	I	R	Hemispheric	10	29
57	48	F	Quad Cane	N	H	R	Hemispheric	38	28
58	48	F	Quad Cane	N	I	BL	Hemispheric	23	28

I, Ischemic; H, Hemorrhagic; L, Left; R, Right; BL, Bilateral; Mixed-Hemispheric and Brainstem Stroke; AFO, Ankle Foot Orthosis; M, Male; F, Female; Y, Yes; N, No.

### Outcome measures

#### Postural Stability

The mCTSIB is an established and reliable instrument for measuring static posture during quiet stance (10, 32, 33). It consists of four conditions designed to measure the relative contribution of each sensory center (visual, somatosensory, and vestibular) in maintaining postural stability during quiet stance in the presence or absence of manipulations to the other sensory systems. The output measure for each mCTSIB condition was the sway velocity index (SVI, degree of sway/s), representing the mean velocity of center of gravity sway during that condition, with higher values representing greater instability.

##### Task

**Figure 1:**
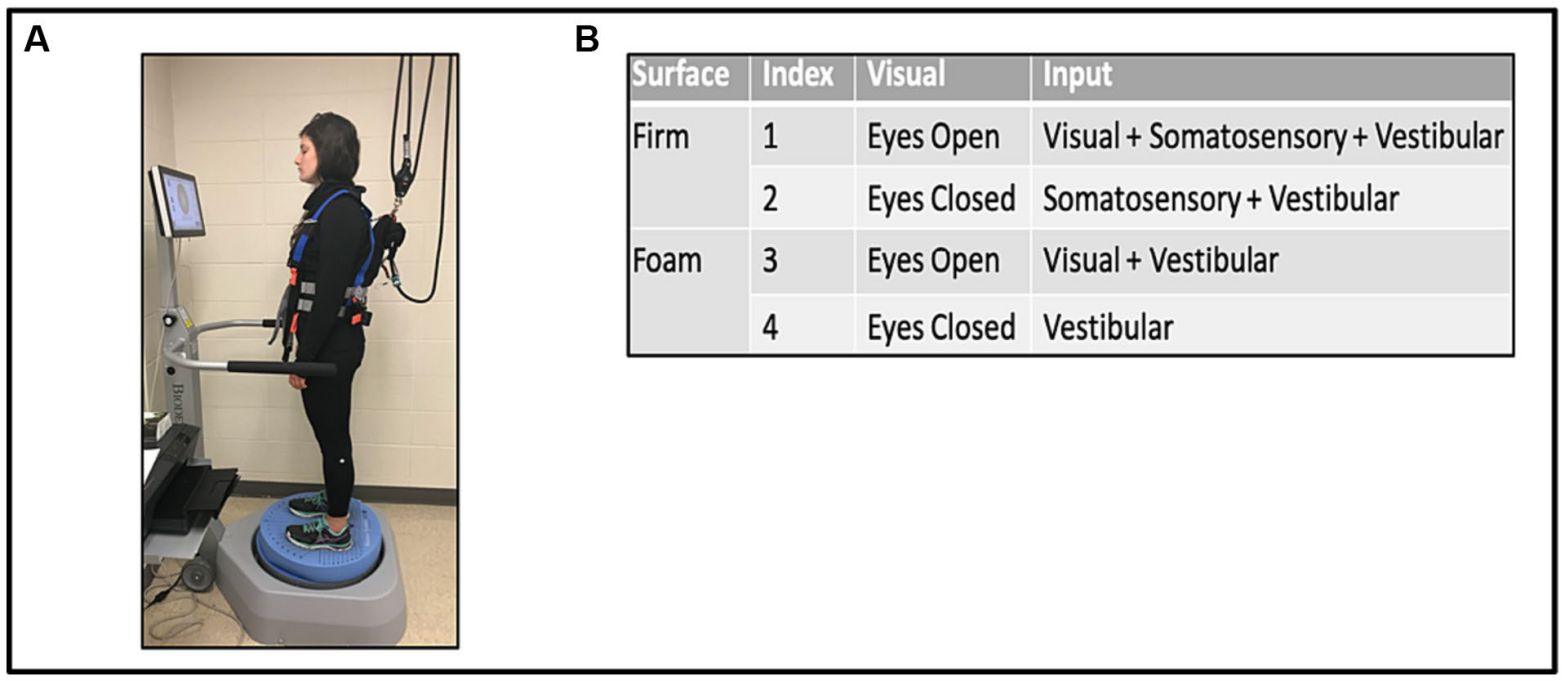
Modified clinical test of sensory interaction in balance (mCTSIB) with the Biodex Balance System™ **(A)**, and the four conditions tested **(B)**.

While on the Biodex Balance Platform™ (Biodex Medical Systems, Shirley NY, United States), with a safety harness and without body weight support or use of balance aids, participants were asked to stand with their hands at their sides, in comfortable double limb stance with shoes approximately shoulder width apart. The test was performed under the four different conditions of the mCTSIB, with each trial lasting 30 s, see Figure 1A. Conditions were: 1 (eyes opened, fixed supporting surface = all sensory modalities are operational); 2 (eyes closed, fixed supporting surface = absence of visual input = somatosensory and vestibular systems unperturbed); 3 (eyes open, foam surface = perturbed somatosensory input = visual and vestibular systems unperturbed); 4 (eyes closed, foam surface = absence of visual input and perturbed somatosensory input = only vestibular function unperturbed; Figure 1B). Participants were allowed to use their prescribed orthosis as a safety measure. Three trials of each condition were performed concurrently and then averaged to determine the mean SVI. In addition, a designation of “non-complete” was given and documented for participants that needed immediate discontinuation or halting of one or more conditions on the mCTSIB due to excessive loss of balance (i.e., falls) or in instances when the side rails were emergently held to avoid falling. Such instances did not generate SVI values and were marked as “non-complete.” Nonetheless, the designation of “non-complete” provided the opportunity to evaluate for potential differences in baseline characteristics and walking capacity between this group vs. stroke survivors who completed all four mCTSIB conditions.

#### Walking speed

The 10-meter walk test (10 MWT) is the gold standard measure of post-stroke walking function reflecting overall mobility and health status (34).

##### Task

A trained physical therapist used a handheld stopwatch to time participants over the 10-meter walkway to obtain the self-selected (SS) and fast-paced (FP) walking speeds. A two-meter space was provided prior to the starting and ending markers to allow for acceleration and deceleration, respectively. The timing started when the participant’s lead leg broke the plane of the starting marker and stopped when both legs crossed the marker at the end of the path. Participants were allowed to use their home assistive device and orthosis. First, participants were asked to walk at their SS speed. Next, they were asked to walk at their FP speed without running. There were two trials for each walking condition, and the respective results were averaged for analysis.

SS walking speed is an universally accepted measure for classifying walking impairment level (household/severe vs. community ambulator/Mild–moderate) (35), while FP walking speed is often associated with a greater risk of imbalance and falls in the elderly (36). We also calculated walking acceleration potential (FP-SS), where a difference of <0.2 m/s has been associated with significant imbalance among chronic stroke survivors (37).

#### Spatiotemporal symmetry during overground walking

Decreased paretic limb contribution to standing balance control has been associated with greater walking asymmetry and increased likelihood of falls due to the limited capacity of the paretic limb to respond to instability (38). Specific to this study, spatiotemporal testing allowed the opportunity to detect differences in spatial and temporal symmetry in walking performance between participants who were able to complete all four conditions of the mCTSIB (i.e., complete) compared with those who were unsuccessful at completing all four conditions (i.e., “non-complete”)—suggestive of insufficient sensory reweighting. Therefore, to test the relationship between insufficient sensory reweighting and lower limb gait symmetry, the average lower extremity single support time (temporal) and step length (spatial) symmetry were collected at the same time as the SS and FP 10 MWT trials using the ProtoKinetics ZenoTM Walkway Gait Analysis System (ProtoKinetics LLC, Havertown, PA, United States). The following equation was used for measuring spatial and temporal symmetry during the 10 MWT: [1 − | Paretic – Non-paretic | / (Paretic + Non-paretic)] * 100%, where the integer values for the respective measure, based on the limb (i.e., paretic or nonparetic) are inputted. Possible symmetry values range from 0% to 100%, where 100% means perfect symmetry and 0% means complete asymmetry (39).

##### Statistics

A total of 58 participants were included in the analysis. The Kolmogorov–Smirnov test was used to assess for deviations from normal distribution among the continuous variables. The means and standard deviations are given, and confidence intervals are reported for all measures. Univariate linear regression using walking speed (variable-dependent), SVI (variable-independent), and non-completion (dichotomous-independent) were performed to determine the relationship between performance on each of the four test conditions and SS and FP walking speeds. The normative SVI value from the general population (*N* = 2,195, Ages: 13–84) provided by Biodex was used for comparison (40). Multiple student t-tests were performed to evaluate differences in SVI between respective conditions on the mCTSIB between our study cohort and normative data, as well as for comparing baseline differences in characteristics between those that were able to complete all four conditions relative to those marked “non-complete” for one or more condition. Chi-square and Fisher’s Exact tests were used only to compare categorical variables between both groups. A significance level was set at *p* ≤ 0.05 for all measures.

## Results

### Postural stability during quiet stance

**Figure 2:**
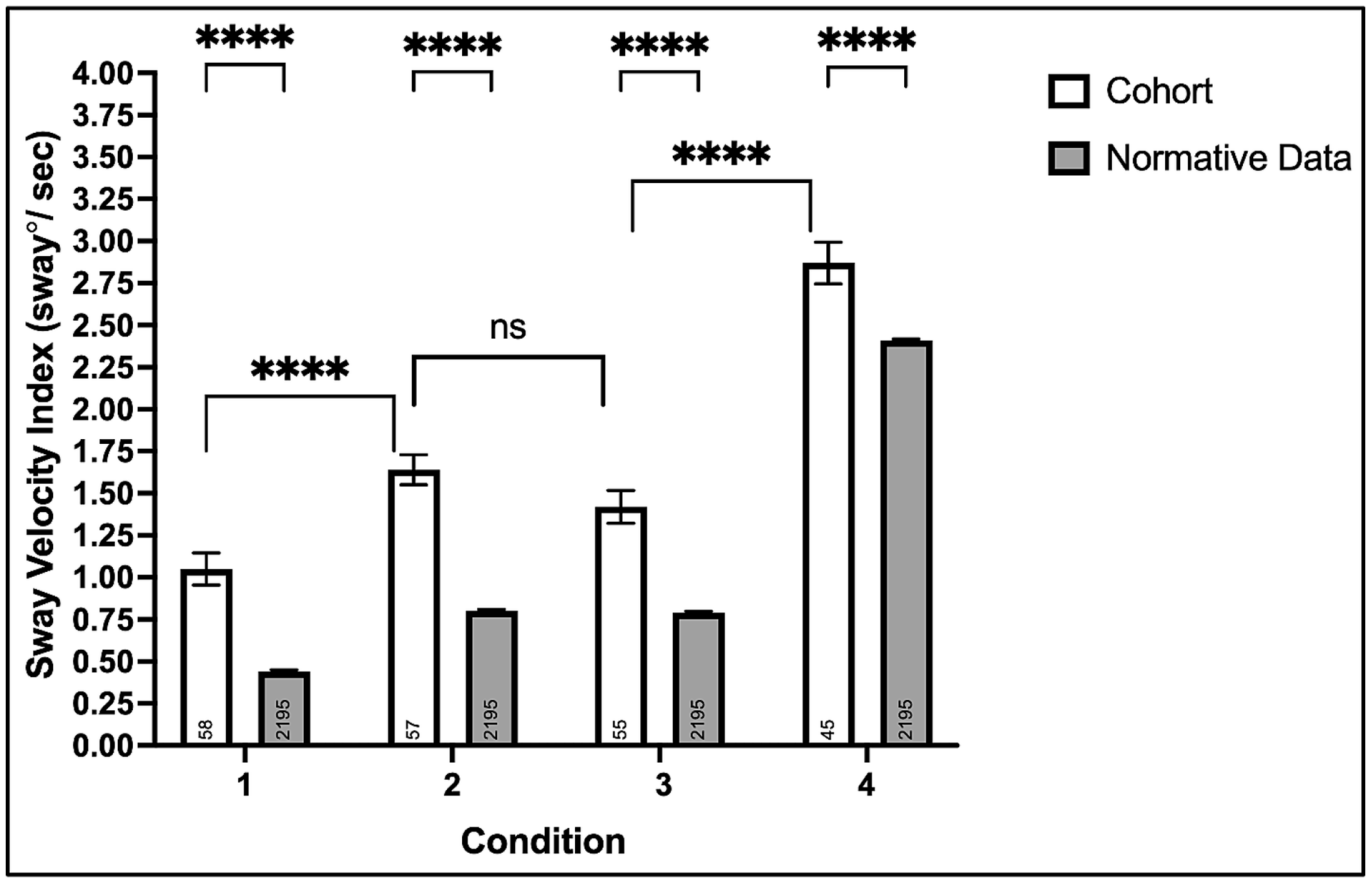
Mean sway velocity index on the modified clinical test of sensory interaction in balance with the Biodex Balance System™. Non-significant (ns, *p* > 0.05) and ^****^p < 0.0001.

*Condition #1*: All 58 participants completed the eyes-opened, fixed support surface condition testing the contributions of visual, somatosensory, and vestibular systems combined on postural stability. Chronic stroke survivors experienced greater SVI (Mean ± SD: 1.05 ± 0.73) compared to normative data (0.44 ± 0.48), *p* < 0.0001 (95% CI: 0.48, 0.74), see Figure 2.

*Condition #2:* Fifty-seven of fifty-eight (98%) participants completed the eyes-closed, fixed support surface condition testing somatosensory and vestibular systems’ contributions to postural balance in the absence of visual input. Chronic stroke survivors experienced greater SVI (1.64 ± 0.67) compared to normative data (0.80 ± 0.44), *p* < 0.0001 (0.72, 0.96).

*Condition #3:* Fifty-five of fifty-eight (95%) participants completed the eyes-opened foam surface condition, testing visual and vestibular systems’ contributions to postural stability when somatosensory information is perturbed. Chronic stroke survivors experienced greater SVI (1.42 ± 0.72) compared to normative data (0.79 ± 0.43), *p* < 0.0001 (0.51, 0.75). Although three participants could not complete condition #3 (compared to only one for condition #2), the SVI was comparable between both conditions, *p* = 0.10 (−0.04, 0.48).

*Condition #4:* Forty-five of fifty-eight (78%) participants completed the eyes-closed foam surface condition testing the contributions of the vestibular system alone and the relative dependence of vision and somatosensation on postural stability. Chronic stroke survivors experienced greater SVI (2.87 ± 0.83) compared to normative data (2.41 ± 0.38), *p* < 0.0001 (0.34, 0.58).

### Walking speed

All 58 study participants completed the walking speed trials. Of these, 25 (43%) required the use of an assistive device during walking (12 cane, 13 quad cane), including 15 who used an ankle foot orthosis (AFO). The average group SS walking speed was 0.62 ± 0.32 m/s and 0.89 ± 0.50 m/s for FP. The average group acceleration potential (FP-SS Speed) was 0.27 ± 0.21 m/s.

Details demonstrating walking speed differences between Completers and Non-completers on mCTSIB, are in Table 2.

**Table 2:** Comparison of baseline characteristics between completers and non-completers.

	Complete (*N* = 45)	Non-complete (*N* = 13)	Value of p
Age (yrs.)	56.5 ± 8.23	58.8 ± 10.4	0.41
Mini-mental status	28.7 ± 2.27	28.4 ± 2.02	0.67
Body mass index	29.0 ± 5.98	27.2 ± 4.51	0.32
Sex			*X^2^* = 0.10, 0.75
Male	22 (49)	7 (54)	
Female	23 (51)	6 (46)	
Stroke type			*X^2^* = 12.8, <0.001^***^
Ischemic	39 (87)	5 (38)	
Hemorrhagic	6 (13.3)	8 (62)	
Location			*X^2^* = 1.27, 0.53
Supratentorial	38 (84.4)	11 (85)	
Infratentorial	4 (8.9)	2 (15)	
Mixed	3 (6.7)	0	
Lateralization			*X^2^ = 1.04, 0.59*
Left	21(46.7)	5 (38.5)	
Right	21 (46.7)	6 (46.2)	
Bilateral	3 (6.7)	2 (15.4)	
Time since stroke (Months)	44.0 ± 51.8	56.2 ± 76.9	0.51
Gait characteristics			
SS speed (m/s)	0.73 ± 0.27	0.23 ± 0.12	<0.001^***^
SS temporal symmetry (%)	85.9 ± 8.74	76.3 ± 12.2	0.002^**^
SS spatial symmetry (%)	90.6 ± 10.8	83.7 ± 7.56	0.04^*^
FP speed (m/s)	1.06 ± 0.43	0.29 ± 0.17	<0.001^***^
FP temporal symmetry (%)	87.4 ± 8.83	75.0 ± 12.8	<0.001^***^
FP spatial symmetry (%)	91.5 ± 9.72	80.1 ± 16.3	0.003^**^
∆ FP speed-SS speed (m/s)	0.33 ± 0.20	0.06 ± 0.07	<0.001^***^
Diabetic neuropathy	5 (11.1)	1 (7.7)	*X^2^ = 0.09, 0.77*
Mobility assistive device			X^2^ = 23.8, <0.001^***^
Cane	8 (17.8)	4 (31)	
Quad cane	4 (8.9)	9 (69.2)	
Orthosis			*X^2^ = 0.02, 0.89*
AFO	12 (26.7)	3 (23)	

Values are presented as mean ± standard deviation with percentage (of total number) in parentheses. Chi-Square score with the corresponding value of p is provided for categorical variables. ^*^p < 0.05, ^**^p < 0.01, ^***^p < 0.001. SS, Self-selected; FP, Fast Paced; AFO, Ankle Foot Orthosis.

### Temporal and spatial symmetry during walking

The average lower extremity temporal symmetry [single support time (s)] during SS and FP walking was 83.7% ± 10.4% and 84.6% ± 11.0%, respectively. The average lower extremity spatial symmetry [step length (cm)] was 89.0% ± 10.5% during SS and 89.0% ± 12.3% during FP walking. Details showing differences in spatiotemporal walking patterns between Completers and Non-completers on mCTSIB, are in Table 2.

### Postural stability and walking speed

The relationship between SVI and walking speed was assessed in participants who completed the posturography task: condition #1 (*n* = 58), #2 (*n* = 57), #3 (*n* = 55), #4 (*n* = 45). There was a weak negative association between SVI in condition #1 and each walking speed: SS [Intercept: 0.75, slope: −0.13 (−0.26, −0.00), *p* = 0.05, R^2^ = 0.07] and FP [1.35, −0.33 (−0.66, 0.00), p = 0.05, R^2^ = 0.07]. Otherwise, there were no associations between SVI in conditions #2–4 and walking speed.

### Non-completion of mCTSIB and walking performance

**Figure 3:**
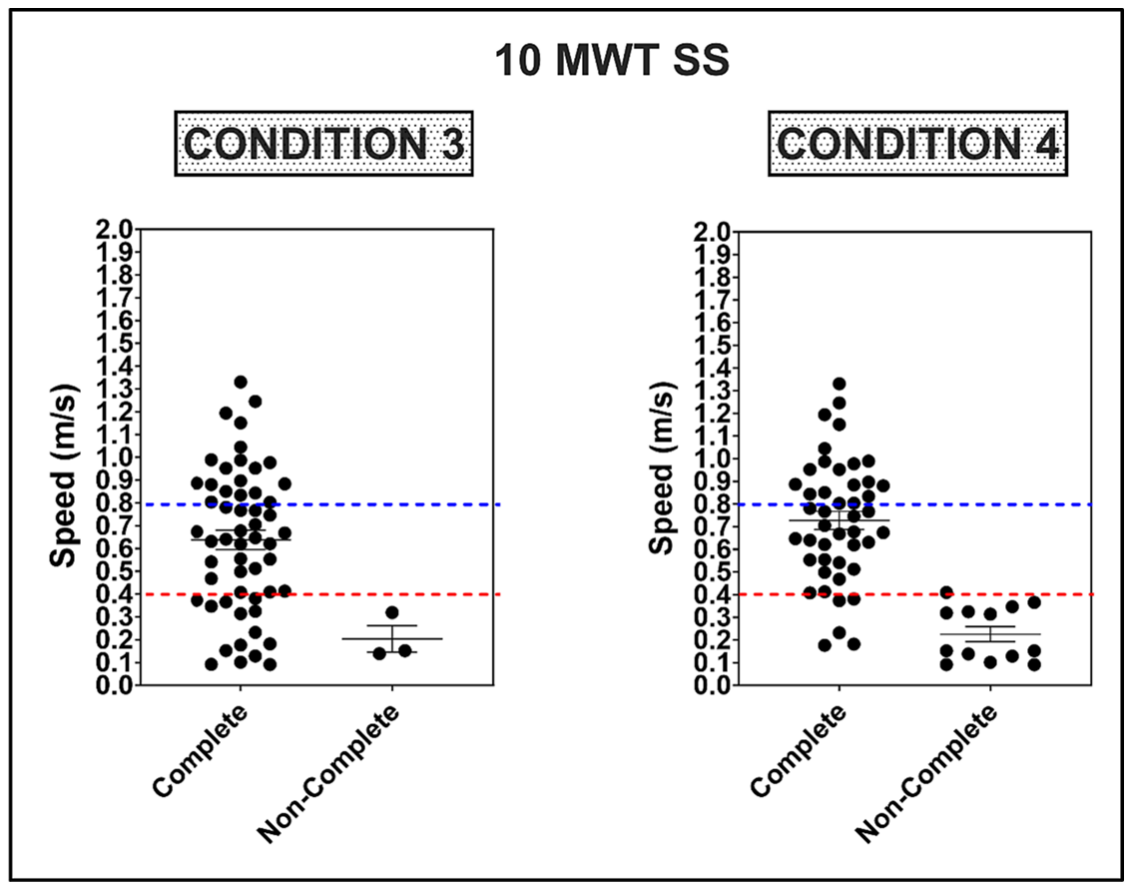
Average self-selected (SS) walking speed on the 10 m walk test (10 MWT) as a function of completing Conditions 3 and 4 on the mCTSIB. Dotted red and blue lines represent the threshold for limited (≥0.4–0.8 m/s) and full community ambulation (≥0.8 m/s), respectively (35).

There was no association between non-completion of condition #2 and overground walking speed [SS: 0.93 m/s, 0.08 (−0.03, 0.19), *p* = 0.14, R^2^ = 0.04; FP: 0.94 m/s, 0.03 (−0.02, 0.12), *p* = 0.15, R^2^ = 0.04]. However, there was a weak association between non-completion of condition #3 and walking speed [SS: 0.81 m/s, 0.21 (0.03, 0.39), *p* = 0.02, R^2^ = 0.10; FP: 0.83 m/s, 0.14 (0.02, 0.25), p = 0.02, R^2^ = 0.09], and a moderate association for non-completion of condition #4 [SS: 0.25, 0.13 (0.59–1.12), *p* < 0.0001, R^2^ = 0.43; FP: 0.29, 0.54 (0.37, 0.72), *p* < 0.0001, R^2^ = 0.42]. The average speed was faster for participants completing all four mCTSIB conditions [0.73 ± 0.27 m/s (SS) and 1.06 ± 0.43 (FP)] compared to participants unable to complete one or more conditions [0.23 ± 0.12 (SS) and 0.29 ± 0.17 (FP)], <0.0001(0.35, 0.66), <0.0001 (0.52, 1.02). Participants unable to complete one or more conditions of the mCTSIB were at or below the established threshold (<0.4 m/s) for self-selected walking speed necessary for community ambulation (*X^2^* = 49.7, <0.0001) (35), see Figure 3. Furthermore, the acceleration difference between FP and SS walking speeds was significantly lower for non-completers (0.06 ± 0.07 m/s) than completers (0.33 ± 0.20 m/s), <0.0001 (0.1563–0.3837). Non-completers also demonstrated more significant spatiotemporal asymmetry during SS and FP walking, see Table 2.

## Discussion

This study aimed to test postural stability during quiet stance and determine differences in visual and somatosensory reliance in our cohort of chronic stroke survivors. We also aimed to determine the relationship between postural stability and overground walking performance. Compared to normative data, we found significantly greater postural instability across all conditions in chronic stroke survivors. In addition, we found an increased reliance on visual and somatosensory systems in chronic stroke survivors, based on comparable SVI values in both conditions 2 and 3 of the mCTSIB, and higher SVI values in condition 4, when both are absent or perturbed, see Figure 2. While there was little to no significant association between the degree of postural instability and walking speed, we report that stroke survivors who had difficulty completing one or more conditions of the mCTSIB were more likely to have severe walking impairment, characterized by SS walking speed less than 0.4 m/s (35). Furthermore, non-completers had strikingly greater walking spatiotemporal asymmetries and diminished acceleration potential. To our knowledge, this study is the first to report the association between performance on specific components of quantitative sensorineural integration testing and overground walking outcomes in chronic stroke survivors. Furthermore, this study is the first to report post-stroke sensory reweighting insufficiency (non-completers) on the mCTSIB and its consequential influence on walking capacity.

In this observational study, all participants were able to complete condition #1 of the mCTSIB, suggesting that the cumulative input of the visual, somatosensory, and vestibular inputs was sufficient to prevent falls during quiet stance in the chronic stages of stroke recovery, irrespective of other factors associated with balance such as cognition, sensorimotor impairment, musculoskeletal strength, and coordination. In addition, there was a small negative association between the SVI and self-selected and fast walking speeds, suggesting that individuals with the greatest SVI under condition #1 are more likely to be slower walkers. We found no association between the SVI and walking speed in conditions #2–4. The absence of this relationship is likely due to the variability in sensory system integrity and implicit strategies used to maintain postural control across study participants. Another possible confounder is that participants with the greatest instability (non-completers) did not generate SVI values for inclusion into the correlation analysis, thereby decreasing the sample size and restricting the distribution of SVI values. Moreover, while postural stability during quiet standing and walking has many similarities, one possibility is that additional biomechanical variables not apparent during quiet stance, such as the shifting of the center of mass and motor activation time, become more relevant during walking (41, 42).

Although there have been conflicting reports on sensory system reweighting in the chronic stages of stroke (14, 26), we found that vision and somatosensation were essential for maintaining postural stability in our cohort—consistent with findings from others in the literature, suggesting that both sensory processing centers can play a key role in post-stroke sensory reweighting (28, 29). This conclusion is supported by the presence of greater SVI in condition #4 (compared with SVI in conditions # 1–3) and the increase in the incidence of non-completions in the absence of accurate visual and somatosensory input. Of note, while it is presumed that the greater SVIs and non-completions were a direct result of the disruption of visual and somatosensory input, an alternate perspective is the post-stroke vestibular hypofunction (43, 44). While none of our study participants had evidence of clinical vestibular dysfunction, previous reports have suggested that stroke survivors with lesions involving the vestibular cortex may experience insufficiency (45, 46). Indeed, the factors driving post-stroke sensory reweighting patterns are complex and likely involve several neuropathologic, neuroplastic, and musculoskeletal variables (24). Uncovering the mediators of inter-individual differences in sensory reweighting patterns in post-stroke is underexplored and warrants further investigation in future hypothesis-driven mechanistic studies.

Interestingly, 88% of our study cohort and 77% of non-completers had supratentorial strokes, as opposed to brainstem and cerebellar strokes—regions classically associated with postural control and balance. Recent reports have shed light on the importance of the human vestibular cortex in motor control, postural stability, and walking outcomes (47–51). Specifically, the parieto-insular vestibular cortex (PIVC) of the human parietal operculum has been suggested to play a vital role in postural control, somatosensory awareness, and central to the function of the vestibular cortex (45, 52, 53). However, its role in predicting balance and walking outcomes after stroke remains unknown. Thus, future studies investigating the PIVC and associated networks as a recovery biomarker may help advance our collective understanding and ability to predict balance and walking recovery outcomes, optimize clinical triaging, and better target rehabilitative interventions.

While task completers and non-completers were similar in characteristics such as age, chronicity of stroke, cognition, and stroke location, we found that the inability to complete one or more of the subcomponents of the mCTSIB was associated with slower walking speeds, greater spatiotemporal asymmetry, and diminished acceleration potential. This finding has both quality of life (54, 55) and rehabilitative implications. Notably, a growing body of work suggests that stroke survivors with severe walking impairment have limited responsiveness to walking rehabilitation interventions compared to survivors with SS walking speeds ≥ 0.4 m/s (55–59)—important considerations for clinical trial design and protocol optimization.

Furthermore, this study found that non-completers were more likely to have had a hemorrhagic rather than an ischemic stroke. However, this finding warrants further investigation in a larger prospective study, as the impact that the stroke subtype (ischemic vs. hemorrhagic) on recovery and rehabilitation remains unsettled (60, 61). Moreover, non-completers were more likely to use walking aids for stability during walking and demonstrated more significant spatiotemporal asymmetry. To this end, the degree of motor impairment, due to its influence on the base of support and stepping reaction patterns during stance and walking (62), may help explain the relationship between non-completion as a result of excessive postural instability (in the absence of somatosensory and visual feedback) and severe walking impairment. Nevertheless, 100% of participants were able to maintain postural stability on Condition #1, and 98% for Condition #2, irrespective of motor impairment status.

Lastly, given possible associations between mCTISB completion and other clinical measures (i.e., walking and clinical balance impairment measures), one may infer that such measures can be used in lieu of the mCTISB. However, the mCTSIB provides valuable and objective information on sensorineural integration and reweighting not attainable with other measures. In addition, mCTSIB with posturography provides invaluable continuous data which can be tracked over the continuum of recovery and used to determine the effects of neurorehabilitative interventions on postural stability and walking outcomes.

### Limitations

Since this study was observational and did not adjust for multiple comparisons, future prospective rehabilitation studies with age-matched controls are needed to validate our findings. Furthermore, while there was no reported or clinically evident vestibular dysfunction in our cohort, formal vestibulo-ocular reflex testing was not performed in this study, therefore the influence of subclinical vestibular dysfunction could not be entirely ruled out and limits the interpretation of our study findings beyond what is reported. Additionally, as designed, the study did not include non-ambulatory stroke survivors; therefore, our findings are not generalizable to that population. Moreover, while this study included many of the standard variables associated with post-stroke balance and walking, additional measures such as lower extremity motor impairment levels, lesion size, structural network integrity of critical pathways involved in sensorimotor integration, and the inclusion of ascending and descending electrophysiological measures may better predict the determiners of outcome. In addition, investigations into how center-of-pressure patterns unfold over time (e.g., nonlinear time-series analysis) may help to elucidate adaptability to changing contextual conditions and transfer between different functional tasks.

## Conclusion

Findings from this cross-sectional study in ambulatory chronic stroke survivors report insufficiencies in sensory systems processing at this stage of recovery and suggest that further consideration should be given to rehabilitation strategies incorporating multisensory system integration testing and strengthening as part of walking rehabilitation protocols. In addition, future walking rehabilitation trials should consider incorporating sensorineural processing measures to evaluate their prognostic potential.

## Data availability statement

The original contributions presented in the study are included in the article/supplementary material, further inquiries can be directed to the corresponding author.

## Ethics statement

The studies involving humans were approved by University of Cincinnati College of Medicine Institutional Board Review. The studies were conducted in accordance with the local legislation and institutional requirements. Written informed consent for participation in this study was provided by the participants’ legal guardians/next of kin. Written informed consent was obtained from the individual(s) for the publication of any potentially identifiable images or data included in this article.

## Author contributions

OA, AG, CD, HS, PB, EW, ME, and KD: conception and design of the study, acquisition of data, or analysis and interpretation of data. OA, HS, SS, PB, AB, KD, PK, and BK: drafting the article or revising it critically for important intellectual content. All authors contributed to the article and approved the submitted version.
